# A Brief Review of Artificial Intelligence in Living Kidney Donation

**DOI:** 10.3389/ti.2025.15334

**Published:** 2026-01-07

**Authors:** Jasir Nawar, Jennifer D. Motter, Jane J. Long, Ritika Sarpal, Dorry L. Segev, Michal A. Mankowski, Macey L. Levan

**Affiliations:** 1 Department of Surgery, NYU Grossman School of Medicine, New York, NY, United States; 2 Department of Computer Science and Engineering, University of California, Merced, CA, United States; 3 Department of Population Health, NYU Grossman School of Medicine, New York, NY, United States

**Keywords:** AI, living donor, living kidney donation, machine learning, kidney transplantation

## Abstract

Artificial intelligence (AI) is rapidly transforming healthcare, and the field of kidney transplantation (KT) is no exception. While much of the AI-related work has focused on deceased donor KT, there is a growing body of research applying AI tools to living kidney donation (LKD). This review explores AI’s current and potential roles in LKD, focusing on predictive and social applications of AI in LKD. Additionally, we discuss the challenges and limitations of implementing AI in clinical settings and highlight emerging research trends. This review consolidates existing research and provides a foundation for both transplant professionals and data scientists seeking to integrate AI responsibly into living donor programs.

## Introduction

Living kidney donation (LKD) remains a vital approach to bridging the gap between supply and demand in kidney transplantation (KT), offering improved graft survival, less delayed graft function, and shorter wait times than deceased donor transplantation [1–3]. Yet, LKD programs confront persistent obstacles: a limited donor pool, intricate immunologic and medical evaluations, and psychosocial factors affecting donor candidacy and retention [1–3].

Artificial intelligence (AI), leveraging modern computational techniques and large-scale data, holds promise for addressing these obstacles. In KT, AI can enhanced risk stratification, optimized organ allocation, and supported recipient management, particularly in deceased donor contexts [4, 5]. However, systematic synthesis of AI’s impact on LKD remains limited.

In this review, we examine current applications of AI in LKD, from risk prediction and donor evaluation to patient education and social media analysis. We evaluate model methodologies and discuss clinical integration, ethical implications, and directions for future research. The goal is to offer clinicians, researchers, and policymakers a clear, evidence-based perspective on AI’s role in advancing living kidney donation.

## Methods

### Literature Search

A comprehensive literature search was conducted across PubMed and Google Scholar to identify studies pertaining to AI in LKD. Search strategies incorporated keywords across two domains: 1) AI and predictive modeling (e.g., generative artificial intelligence, machine learning), and 2) living kidney donation (e.g., live kidney donation, living kidney donor, living donor kidney transplantation [LDKT]). We excluded studies that were abstracts without full text, non-English publications, or focused on deceased donation in their methods. Only articles that were published from 2008 onward were considered to ensure a focus on contemporary techniques and advancements. Study selection was finalized through iterative discussion among JN, MM, and ML (Table 1).

**TABLE 1:** Summary of AI applications in living kidney donation.

Tool name	Model(s) used	Population	Application	Results	References
Traditional machine learning models					
KNN + naïve bayes	Naïve bayes + KNN	n = 2728	Graft failure prediction (5 years)	80.77% accuracy 73.5% F1 score	Atallah et al. [6]
UK-LTOP	XGBoost	n = 12,661	Graft failure prediction (1–12 years)	5 years: 0.73 AUROC, 0.72 C-statistic 10 years: 0.75 AUROC, 0.72 C-statistic 12 years: 0.79 AUROC, 0.72 C-statistic	Ali et al. [7]
KDNI	XGBoost	n = 823	Post-donation eGFR (6–12 months)	0.900 AUROC, 73.2% sensitivity, 90.3% specificity	Jeon et al. [8]
Neural networks					
Neural network	Neural network	n = 1,900	Graft survival prediction (5 years)	0.88 AUROC, 88.43% sensitivity, 73.26% specificity, 88% accuracy, 82.1% PPV, 82% NPV	Akl et al. [9]
3D DenseNet	DenseNet	n = 1,074	Post-donation eGFR	Donors with higher remnant kidney volume to weight ratio exhibit less change in post donation eGFR	Jo et al. [10]
CNN	CNN	n = 1,930	Segmentation of kidney CT scan and estimation of kidney volume	p > 0.05 for all estimated volumes of cortex and medulla	Korfiatis et al. [11]
LSTM	LSTM	n = 203,219	Classification of social media post relation to LKD	60.2% F1 36.8% specificity 77.1% sensitivity 62.9% accuracy	Asghari et al. [12]
Transformer models					
ChatGPT 3.5, BERT	Transformers	n = 3292	Classification of reddit post relation to LKD	78% F1 79% specificity 79% sensitivity 79% accuracy	Nielsen et al. [13]
ChatGPT, MedGPT, gemini	Transformers	n = 35	Evaluation of readability of generated information on LKD	39.42 FRES 10.63 FKGL	Villani et al. [14]
ChatGPT 3.5, ChatGPT 4.0	Transformers	n = 27	Evaluation of readability of generated information on LKD	Reduced FKGL by 4.30 ± 1.71 (p < 0.001) 96% reduction of information to eight grade level or below	Garcia Valencia et al. [15]
ChatGPT	Transformers	n = 20	Evaluation of accuracy of generated information on LKD	55% of responses rated to have ≥80% accuracy 85% of responses rated to have ≥66% completeness 85% of responses rated to be ≥75% not harmful	Xu et al. [16]
Specialized models					
L-TOP	XGBoost + deep cox mixtures	n = 66,914	Graft failure prediction (1.5–13 years)	5 years: 0.70 AUROC, 0.70 CTD 10 years: 0.68 AUROC, 0.67 CTD 13 years: 0.68 AUROC, 0.66 CTD	Ali et al. [17]
RAPTO	AutoScore	n = 823	Post-donation eGFR (6–12 months)	0.846 AUROC, 0.965, AUPRC	Jeon et al. [18]
Gia chatbot	Decision tree chatbot	n = 54	APOL1 risk education	82% agreed “neutral and unbiased”, 82% agreed “trustworthy”, and 85% “words, phrases, and expressions are familiar to the intended audience”	Gordon et al. [19]

APOL1, apolipoprotein 1; AUROC, Area Under the Receiver Operating Characteristic Curve; AUPROC, Area Under the Precision-Recall Curve; CNN, convolutional neural network; CTD, time-dependent concordance index; eGFR, estimated glomerular filtration rate; FKGL, Flesch–Kincaid Grade Level; KDNI, kidney donor network initiative ;KNN, k-nearest neighbors; LKD, living kidney donation; LSTM, Long Short-Term Memory; PPV, positive predictive value; NPV, negative predictive value; RAPTO, Risk Assessment Post-donation Tool Using Outcome-based AutoScore; UK-LTOP, Live-Donor Kidney Transplant Outcome Prediction tool, XGBoost, Extreme Gradient Boosting.

## Overview of AI Methods in LKD Research

### Common Metrics to Assess Model Quality

The studies discussed throughout this review used common machine learning metrics to assess the performance of their models with respect to accuracy, discrimination, calibration (Table 2).

**TABLE 2:** Common metrics to assess model quality.

Metric	Definition	Clinical interpretation
Sensitivity or recall	Proportion of true positive cases correctly identified by the model [20]	High sensitivity indicates few patients with the condition are missed. This is important for screening or early detection, where failing to identify a case may have deleterious consequences
Specificity	Proportion of true negative cases correctly identified by the model [20]	High specificity minimizes false alarms. This is important for confirmatory testing or when unnecessary treatment should be avoided
Positive predictive value (PPV) or precision	Proportion of predicted positives that are truly positive [20]	High PPV indicates that a positive result reliably suggests that the patient has the condition. This is important when providers rely on positive results to guide diagnosis or initiate treatment; PPV strongly depends on prevalence of the condition
Negative predictive value (NPV)	Proportion of predicted negatives that are truly negative [20]	High NPV indicates that a negative result reliably suggest that the patient does not have the condition. This is important in ruling out the presence of the condition; NPV strongly depends on the prevalence of the condition
F1 score	Harmonic mean of precision and recall [20]	High F1 indicates that the model achieves good balance between false positives and false negatives. Ignores true negatives; may be clinically misleading when false positives carry a substantial burden. This is important when both types of diagnostic errors have clinical consequences (e.g., missing a diagnosis and over-diagnosing are equally undesirable)
Area under the receiver operating characteristic curve (AUROC)	Measures a model’s ability to discriminate between patients with and without the condition across all thresholds. AUROC values range from 0.5 (0.5 is random chance) to 1.0 [20]	High AUROC indicates that the model can reliably discriminate between patients with and without the condition, independent of the specific decision cutoff. Disscrimination only; does not indicate PPV/NPV at a working threshold or calibration
Area under the precision-recall curve (AUPRC)	Measures the tradeoff between precision and recall across all thresholds. AUPRC values range from 0 (poor performance) to 1.0 (perfect performance) [20]	High AUPRC indicates that the model correctly identifies most patients with the condition while rarely misclassifies patients without the condition as positive. Baseline depends on prevalence; especially relevant for imbalanced outcomes. This is important when the disease is rare, and both false positives and false negatives can be costly

### Overview of Clinical Problems and Models

We provide an overview the clinical problems addressed in the included studies, the models used to address each problem, and the rationale for the usage of certain models (Table 3).

**TABLE 3:** Key clinical challenges in living kidney donation and potential AI solutions.

Clinical challenge/Question	Models used	Rationale
Transplant outcome prediction	• Naïve bayes • KNN • XGBoost • Neural network • Deep cox mixtures • AutoScore	• Naïve bayes and KNN are simple methods which can fetch modest results with some tuning. They can also be used as supporting methods in data preprocessing • XGBoost, neural networks, deep cox mixtures, and Autoscore can have very good results in prediction at the cost of some added complexity
Education	• LSTM • Transformers • Decision tree	• LSTM and transformers are well suited in identifying long term dependencies in text sequences • Transformers-based models are currently the state of the art in on-the-fly text generation • Decision trees provide an easy to interpret hierarchy which can be easily explained
Donor risk or perioperative risk	• Deep cox mixtures • Autoscore	• Currently, a deep cox mixtures model has had its results compared to LKDPI • Autoscore is a framework to build customizable risk scores
Image analysis	• CNN • 3D DenseNet	• The internal computation of CNNs and 3D DenseNet, which is called the “convolution” is and has been well established in analyzing image data

### Traditional Machine Learning Models

Traditional machine learning (ML) techniques such as eXtreme Gradient Boosting (XGBoost), K-Nearest Neighbors (KNN) algorithm, and Naïve Bayes (NB) algorithm have been used to create post-operative prediction systems of outcomes such as graft survival and post-operative renal insufficiency. These models aim to better inform LKD donors about their donation risks and support clinicians in counseling potential LKD donors.

XGBoost is an ensemble-learning method that combines multiple smaller models into a single, more accurate one. This is done by building many shallow decision trees sequentially, each one improving upon the errors of the last [21]. The model focuses on efficiency, speed, and high performance, using parallel processing to train models on large datasets. It is a highly effective model for training on tabular clinical datasets across various prediction tasks due to its handling of missing data, ability to automate ranking of variables, and regularization to reduce overfitting, which can reduce model training time compared to manual methods [21]. XGBoost has demonstrated versatility across multiple applications in LKD research. XGBoost was employed for variable selection in the Live-Donor Transplant Outcome Prediction model (L-TOP), leveraging its ability to handle missing values for automated elimination of variables [17]. In the UK, researchers used XGBoost alone to develop a predictive model for graft failure in LKD recipients using data from the UK Transplant Registry, where it was favored due to the presence of nonrandom missing data. Compared to a decision tree approach and a random survival tree approach, XGBoost provided the highest Area Under the Receiver Operating Characteristic (AUROC) score for all time points of its task [7]. In other words, across all time points, the XGBoost based model achieved and maintained the highest ability to distinguish between LKD patients with graft failure and those whose graft survived. Additionally, XGBoost has been applied to predict post-donation eGFR of donors in order to identify individuals at risk for post-donation renal insufficiency [8]. The XGBoost-based model achieved the highest AUROC and showed the strongest correlation between predicted and observed pre- and post-donation eGFR values. These findings suggest that the model may offer a reliable tool for forecasting postoperative eGFR outcomes, potentially assisting clinicians in the evaluation and selection of living kidney donors [8].

XGBoost outcomes are less interpretable than commonly used linear models due to modeling and training methods involving multiple decision trees. One method to create a more interpretable model is to combine multiple, more interpretable algorithms together. In 2019, Atallah et al. combined two model, KNN and NB, to create a model for measuring five-year graft survival of LKD recipients [6]. KNN is a predictive model that categorizes a new sample into a class by identifying the ‘k' closest samples and assigning it the most common class among them, making it far more interpretable than XGBoost since model decisions are due to proximity to groups of samples [22]. However, since KNN is directly dependent on the number of samples, its performance degrades as the amount of data, number of variables, or dimensionality increases.

Atallah et al. addressed this by using a Naïve Bayes (NB) algorithm for variable selection, identifying the most relevant inputs for KNN based on probabilistic categorization [6]. Naïve Bayes (NB) is a probabilistic model that assumes independence among variables yet often performs well in medical contexts where interpretability is critical, such as disease and risk prediction [23]. Atallah et al. used NB to iteratively exclude individual variables, retaining those whose removal reduced model accuracy [6]. Given the need to evaluate 44 variables, this approach leveraged NB’s computational efficiency. The combination of NB for variable selection with KNN ultimately demonstrated the highest accuracy and mean F1 score compared with nomogram, decision tree, Bayesian network, and neural network models available at the time [6].

Traditional ML techniques are already well established and continue to remain valuable for developing predictive models in LKD. With the availability of more powerful computational hardware, combining multiple ML algorithms in one model has become increasingly easier to do. As these combined models are composed of multiple, interpretable pieces, these models can potentially improve predictive performance, while maintaining the interpretability needed for clinical decision-making.

### Neural Networks

Neural Networks (NN) are being used to gather insights from high-dimensional inputs where regular variable selection is not feasible. NNs are used to mimic the decision-making manner of the human brain and consist of layers of interconnected nodes acting akin to connected neurons [24]. Basic neural networks are early examples of deep learning, capable of capturing complex nonlinear patterns. Deep learning is a natural evolution of early NNs, where more layers of nodes could be trained to capture far more nuanced patterns due to increased computational ability in the past decade [25]. Early predictive models in LKD include efforts to use basic NNs for five-year survival estimation [9]. In a study by Akl et al., NNs were trained on donor-recipient variables to learn patterns associated with successful transplant outcomes [9]. While less complex than modern NN models, this work demonstrated the early potential of in LKD graft survival prediction. Compared to nomogram-based models to predict 5-year graft survival estimation, the NN achieved higher sensitivity and predictive accuracy, and achieved almost twice the positive predictive value [9].

Convolutional neural networks (CNNs) are a type of neural network designed to process image data through convolutional layers that analyze each pixel in the context of its neighbors, enabling effective detection of edges and textures. CNNs are used in facial recognition, object detection, and handwriting recognition [25]. In a study by Korfiatis et al, CNN-based segmentation was used to quantify cortical and medullary kidney volumes from imaging data [11]. The results showed correlations between segmentation with clinical donor characteristics, offering a new biomarker for renal health and transplant planning [11]. Additionally, the segmentation of kidney CTs were achieved in less than 5 min, a great increase in efficiency compared to the 30–90 min that a human observer could take on the same task [11].

Densely Connected Convolutional Network (DenseNet) is a deep CNN designed for efficient image classification. Unlike other NNs in which each layer shares its output with the next layer, DenseNet connects each layer to every other layer in a feedforward fashion. This improves parameter efficiency and information flow, preventing variables from being lost or ignored [26]. Applications of DenseNet include object recognition for autonomous driving, X-ray analysis, and robot vision. A 3D DenseNet model was developed by Jo et al. to measure kidney volume from CT images in elderly donors [10]. Viewing the model’s measured kidney volume against post-donation eGFR revealed significant negative correlations in elderly donors [10].

In general, CNNs have already been applied effectively in image-based LKD prediction tasks. However, despite the strength of NNs to easily deal with high dimensional inputs, less sophisticated techniques with lower variables may be favored for interpretability, especially regarding prediction modeling such as predicting LKD outcomes.

### Language Models

Language models are models which specifically process text as input data for tasks. At the time of this writing, language modeling has exploded as one of the most popular areas in the field of AI, particularly due to the creation of transformer models [27]. From this newfound popularity of transformers, text-based tasks in LKD such as donor outreach and communication have enjoyed a recent surge of interest as well [28]. While transformers may be popular now, previous models such recurrent neural networks (RNN) can be relevant to language tasks where simplicity and lower computational cost are a concern.

#### Transformer Models

Transformer models use self-attention mechanisms to evaluate all words in a sentence simultaneously, identifying key relationships regardless of word position or distance [29]. Bidirectional Encoder Representations from Transformers Models (BERT) consider both previous and subsequent words to better understand the input text [30]. Meanwhile, Generative Pretrained Transformer Models (GPT) generate human-like responses based solely on previous words [27]. BERT models focus on reading comprehension and classification tasks such as search engines and text classification. GPT models focus on writing, chatting, and summarizing and have applications in text generation and translation. A notable aspect of transformer models is the ability to fine-tune them, which involves taking a transformer model previously trained on a general corpus of information and training this pre-trained model on a dataset specific to some tasks, essentially “specializing” the model to the desired task [30]. Nielsen, et al., 2025 fine-tuned BERT and GPT models and used them to determine if Reddit posts were written by users who presently undergoing experiences in LKD, users who previously experienced effects of LKD, and general LKD news [30]. Gathering these insights into donor anxiety, medical concerns, social support needs, and emotional experiences at scale has only just become possible with current transformer models [13].

In educational applications, GPT-based models have been evaluated for generating materials on LKD, demonstrating the ability to produce accurate and readable content at the college level for public awareness campaigns and patient education [14]. ChatGPT has shown promise in improving patient communication by rewriting FAQs for living donors, where it was found to significantly reduce the reading level of FAQs, enhancing clarity for users with diverse literacy levels [15]. Additionally, ChatGPT’s responses to real patient questions about kidney transplantation have been analyzed for accuracy, completeness, and potential harm and has demonstrated the ability to provide accurate and up to date information for most LKD questions which are commonly asked, suggesting its potential as a valuable supplement to human education efforts [16]. However, limitations from model “hallucinations”, or the generation of responses which are factually and logically incoherent, remain [31].

#### Recurrent Neural Networks

RNNs process sequential data retaining previous steps in memory in order to understand current tasks. However, important events from less recent steps are often forgotten. Long Short-Term Memory models (LSTM) are a type of RNN that better retain relevant information across longer time steps, making them well-suited for modeling long-term patterns such as the general populace sentiment with respect to LKD. LSTMs use gating mechanisms which filter events that should be kept, used, and forgotten [32]. Applications of RNNs and LSTMs include autocomplete during typing, speech recognition such as Siri or Google Assistant, and language translation. Asghari et al., 2022 used a deep RNN using LSTM nodes to classify and interpret social media posts, reliably determining whether they were related to LKD [12]. This approach demonstrates the potential for automating the identification of potential LKD donors on a large social media scale [12].

#### Rule-Based Methods

Despite the very recent advances in language modeling, well designed simple models still work well for very specific tasks while also offering high interpretability for clinicians. Rule-based chatbots occupy this niche, as they rely on decision trees or if-then logic to guide users through structured interactions. They are designed to educate and support patients in their choices. Although they are not as sophisticated as chatbots driven by GPT based models, these models offer greater transparency and consistency. The “Gia” chatbot, a rule-based conversational agent, was developed to educate African American donor candidates about the APOL1 gene, a known genetic risk factor for kidney disease. In testing, users generally found the chatbot to be neutral, unbiased, and trustworthy [19]. Until the extent of racial bias with respect to transformer models is extensively studied and addressed, simple language models such as Gia are best suited to tackle LKD language tasks targeted at specific populations.

Current transformer models and prior RNNs have made it possible for researchers to evaluate how LKD patients respond to their care at a scale previously unimaginable. At the same time, GPT-based models have made it possible to communicate with patients in a new way, creating LKD content automatically and with reasonable accuracy.

### Specialized Models

In some studies, some customized models were developed in an attempt to address LKD problems in a more unique way compared to previously discussed models. There are currently few specialized AI architectures which have been deployed to address LKD problems. However, the studies done suggest potential upsides for these approaches in LKD outcome prediction and even building new profiling methods in LKD.

One type of specialized model is Deep Cox Mixtures (DCM), where NNs are used to generate patterns from input patient data and then a combination of Cox models is then used to predict time-to-event outcomes based on the generated NNs patterns. This method enables personalized, flexible risk estimation and uses hazard functions to retain interpretability [17]. Applications of DCMs include survival estimation of various diseases, such as cancer, as well as variables associated with diseases, such as serum free light chain, for example. DCM was incorporated into the Live-Donor Transplant Outcome Prediction (L-TOP) model to predict death-censored graft failure of LKD recipients. The predictive ability of L-TOP outperformed the live kidney donor profile index on the same population [17]. At the cost of some complexity, DCM was able to elevate the bar for donor profiling.

In addition to elevating clinical scoring, specialized models open the possibility for creating clinical scores. AutoScore is a specialized model that combines multiple models for variable selection, discretization, and logistic regression to produce interpretable risk scores that facilitate clinical decision-making [18]. Autoscore translates the outputs of a combination of multiple models into a simple, point-based scoring system. In LKD, AutoScore has been used to develop models that stratify donor risk based on readily available preoperative factors such as demographic information, kidney volume, GFR, and lab values [10]. Jeon et al. used the AutoScore model to build an interpretable scoring system for renal adaptation, allowing clinicians to stratify donor risk using a simple point-based system [33]. The generated scoring model was able to predict the probability of fair renal adaptation, defined by the study as a post-donation eGFR ≥60 mL/min/1.73 m^2^ [33]. Prior to Jeon et. al, few studies existed to predict renal adaptation based on pre-operation variables, let alone a clinical score for renal adaptation. The Autoscore model leverages multiple interpretable and generalizable modules as part of its architecture, potentially allowing risk scores to be generated for other LKD outcomes.

## Discussion

Recent developments indicate a shift toward more comprehensive, personalized, and interpretable applications of AI in LKD. Emerging work aims to integrate multiple AI technologies for more robust decision-making, while also addressing ethical, social, and clinical concerns associated with transplantation.

One major trend is the prevalence of simpler AI architectures in clinical tools. More advanced, modern transformer-based and deep learning architectures often function as “black boxes,” delivering predictions without clarity on their reasoning [34]. While explainability techniques such as SHAP (SHapley Additive exPlanations) can be used to address this ambiguity; however, it is important to note that still there are limitations to consider when it comes to SHAP in its ability to explain models [34]. To address this challenge, approaches in LKD literature, Atallah et. al., AutoScore and L-TOP aim to balance accuracy with interpretability [6, 17, 33]. These models prioritize transparency, enabling clinicians to understand the rationale behind each prediction, which in turn fosters trust and supports shared decision-making.

Another key trend is the use of large electronic health records (EHRs) datasets to improve generalizability across populations. As highlighted recently, efforts are being made to overcome single-center biases by training models on national data [35]. National and representative data help mitigate the risk of algorithmic bias, ensuring that AI tools perform reliably across diverse demographic and geographic settings.

Finally, AI is increasingly seen not only as a predictive engine, but also as a communication facilitator. Tools like GPT-based chatbots are being adapted to support personalized donor education, provide real-time patient support, and enhance counseling.

### Challenges and Limitations

#### Data Heterogeneity

Despite its promise, the application of AI in LKD faces several persistent challenges. One of the foremost barriers is the heterogeneity of available data. Clinical datasets often vary in format, quality, and completeness across institutions. This results in situations where, for the same problem, certain approaches are non-transferrable due to differences in data collection processes and variable availability, as observed in LTOP and UK-LTOP [7, 17]. Moreover, data missingness within collected variables can create additional challenges for model development and interpretation. While imputation techniques can address missingness in certain contexts, non-random missingness makes imputation inappropriate, as it introduces bias. This constraint influenced model selection in UK-LTOP, where tree-based algorithms were selected over DCM (successfully used in L-TOP) as they handle missing data without imputation. Furthermore, variations in imputation strategies affect evaluation metrics, particularly the AUROC score, which may impact performance comparisons and limit external validity.

#### Validation

In addition to the heterogeneity of available data, the current lack of scale poses a substantial obstacle. At present, the largest dataset that evaluated LKD transplant outcomes was limited to tens of thousands of patients, with test samples of similar size [17]. Lack of scale and diverse cohorts not only hinders the development of robust models, but it also prevents adequate large-scale external validation–an essential step in establishing generalizability and adoption of prediction models [28, 36, 37]. Although LKD models may report promising results, especially in predicting donor outcomes, their broader applicability is uncertain. It is difficult to ascertain whether a model is prone to overfitting to specific patterns without validating it using data beyond the test set of a study, even when techniques like cross-validation or temporal splits are employed. Therefore, it becomes unclear how susceptible the model is to data drift (i.e., changes in the distribution of input data over time as new data is incorporated) [36]. Along with the need for large-scale external testing, few of the discussed studies compared the predictive performance of their models to current standard models, such as the living kidney donor profile index (LKDPI) [17]. While this comparison may not be relevant for all studies, it would be valuable for graft survival prediction models to understand the benefit of the proposed techniques. Overall, robust validation methods external testing in diverse cohorts are essential to ensure consistent and reliable performance.

#### Model Complexity

There is an ongoing tension between model complexity and overfitting, which underscores the challenge of balancing model accuracy and generalizability. Rooted in the bias-variance tradeoff, greater model complexity increases the risk of achieving high performance on inputs that are specific to the training data, but fails to generalize effectively to unseen test data or real-world clinical settings. Additionally, increased model complexity often comes at the cost of reduced explainability, which can introduce undue risk for providers in decision-making. As such, simpler models may be preferred to prevent this from occurring. However, in some instances, such as images or large-scale data, a more complex model is often better suited to capture the increased complexity and scope. When applying complex models to clinical decision-making, it is essential to assess their external validity, calibration, and decision-support usefulness. This can be achieved using standard performance metrics (Table 1) alongside evaluations of model transportability and integration into clinical workflows. Ultimately, appropriate model selection requires careful evaluation of the input data, goals, and tradeoffs, ensuring the design of a model that balances performance with explainability and real-world applicability.

#### Adoption

Despite rigorous validation, numerous barriers persist in the clinical adoption of AI. One key barrier is inconsistent reporting of metrics across models, particularly in outcome prediction models. Standardized reporting of performance metrics (Table 1) would enhance comparability and help identify which models are most appropriate for decision support in clinical use.

Another important barrier is the lack of trust in predictive modeling. Providers may be hesitant to rely on models that do not have clear explainability or transparency, especially in the current context of minimal regulatory oversight [38, 39, 40]. Addressing concerns around liability from inaccurate predictions is important for clinical adoption and emphasizes the need for regulations.

Ethical issues also arise when AI tools are used in patient-facing applications like donor or recipient education and social media analysis. Consent, bias, and data safety are key ethical issues that must be considered for the responsible and equitable use of AI tools [28, 36, 39–41]. Experiments involving AI models like ChatGPT require more consideration of their results, as the datasets used for evaluation are often curated from online sources such as community-generated benchmarks and platforms aggregating human activity [42]. This introduces questions about the provenance of data, quality control, and the need for inclusion of diverse perspectives, particularly when vulnerable populations are involved.

Establishing standardized benchmarks to assess these tools, particularly in scenarios such as LKD communication, remains an important challenge. Evaluating AI tools for fairness, especially when vulnerable populations are involved, remains equally important [4, 28, 37, 39]. Addressing these concerns requires robust standards for ethical AI implementation in clinical environments, which will be essential for promoting trust, safety, and equitable outcomes in patient care.

Finally, issues of cost must be addressed when considering integrating AI into clinical practice. Infrastructure requirements like data storage, data maintenance, and security add significant expenses. From a modeling perspective, substantial costs arise from training predictive models and deploying them across health systems, which is necessary to ensure quick, reliable, and easily accessible model outputs for clinical decision-making.

## Conclusion

AI is beginning to play a meaningful role in LKD, from predicting outcomes and improving donor-recipient matching to analyzing social media and enhancing patient education. Still, most models require validation with multicenter data, and future work should prioritize interpretability. Usability and fairness must also be addressed to ensure these tools can be effectively and equitably integrated into transplant care. As this field grows, close collaboration among clinicians, data scientists, and ethicists will be essential to realize the full benefits of AI in LKD.
